# Norwegian translation, cultural adaption and testing of the Person-centred Practice Inventory – Staff (PCPI-S)

**DOI:** 10.1186/s12913-018-3374-5

**Published:** 2018-07-16

**Authors:** Pia Cecilie Bing-Jonsson, Paul Slater, Brendan McCormack, Lisbeth Fagerström

**Affiliations:** 1 University of South-Eastern Norway, Postbox 23, 3603 Kongsberg, Norway; 2 University of Ulster, York Street, Co. Antrim, Belfast BT15 1ED UK

**Keywords:** Person-centred, Questionnaire, Measurement, Translation, Cultural adaption, Health care staff

## Abstract

**Background:**

Person-centred health care has widespread recognition, but there are few instruments aimed at measuring the provision of person-centred practice among health care professionals across a range of settings. The Person-centred Practice Inventory – Staff (PCPI-S) is a new instrument for this purpose, theoretically aligned with McCormack & McCance’s person-centred framework, which has been translated and culturally adapted into Norwegian.

**Methods:**

The study used a two-stage research design involving: translation and cultural adaption of the PCPI-S from English to Norwegian language (phase 1), and a quantitative cross sectional survey following psychometric evaluation (phase 2). Confirmatory factor analysis was used to examine the theoretical measurement model.

**Results:**

The translation and cultural adaption was carried out according to ten recommend steps. Discrepancies were addressed and revised by all translators until consensus was reached on a reconciled version of the translation. A sample of 258 health care staff participated in the survey. The model fit statistics were overall positive; the model requires minor modifications and these are mostly confined to correlated errors.

**Conclusions:**

The translation and cultural adaption process of the PCPI-S from English to Norwegian language was a demanding process in order to retain the conceptual meanings of the original instrument. Overall, the psychometric properties of the tool were acceptable, but testing on a larger sample size is recommended.

**Electronic supplementary material:**

The online version of this article (10.1186/s12913-018-3374-5) contains supplementary material, which is available to authorized users.

## Background

### Person-centred health care; philosophy and measurement

Person-centredness has become a global movement in healthcare. With a focus on creating cultures that place ‘the person’ at the centre of decision-making, person-centredness is a philosophy that underpins many western healthcare policy positions and strategic developments. Whilst the evidence underpinning person-centred healthcare continues to be diverse, using multiple terms to capture the key components of person-centred practice, there is increasing consensus about the key principles underpinning person-centredness. These principles underpin a range of emerging models and frameworks used to guide practice, organisational and strategic developments, with a focus on creating more person-centred cultures in healthcare systems. In this paper we focus on one such framework [1] and its application to practice. We present an overview of the framework and then focus on the translation, cultural adaptation and testing of an instrument developed to evaluate the extent to which person-centred cultures are developed using the framework of McCormack and McCance [1] as a guide.

The theoretical framework of person-centred health care provided by McCormack and McCance [1] outlines the following key aspects of the concept: being in social relationships; being in a social world; being in a physical place; and being with self. A central premise in the theory is the development of therapeutic relationships between service users and health professionals. The therapeutic relationship is underpinned by values of respect for the person, individual right to self-determination, mutual respect and understanding [1]. The prerequisites address the attributes of staff that must first be considered, in the context of developing an effective care environment. The person-centred processes identify key practices that underpin effective person-centred care services. However, the framework also recognises the complexity of organisational systems and the challenges these pose in developing person-centred care [2], which can be explored and better understood by comprehensive measurement of the person-centred attributes of staff.

Person-centred health care has widespread recognition worldwide, and the concept has also been spread to the Nordic countries Sweden and Norway [3]. In a critical review of how to measure person-centred care, Edvardsson and Innes [4] stated that a common approach in studies of person-centred interventions has been to use outcome measures as proxy descriptors of person-centeredness, and found that until recently person-centeredness was not measured as a concept per se. A literature search revealed, however, that there are numerous instruments measuring person-centeredness today. The Health Foundation [5] identified the existence of 176 instruments to measure person-centredness. However most of these are proxy measures and are focused on patient-outcomes. Edvardsson and Innes [4] found and reviewed twelve instruments for person-centred care for older people and people with dementia, eleven developed for patients and one for staff. Köberich and Farin [6] reviewed three instruments measuring person-centred nursing care from the patient’s perspective. Two person-centred instruments have been translated into Norwegian and have been evaluated for psychometric properties; the Person- centred Climate Questionnaire–Staff version (tested with nursing home and hospital staff) [7], and the Person-centred Care Assessment Tool (tested with staff working in residential units for older people) [8].

DeSilva [5] found that although there are instruments developed for specific settings like nursing homes, there are few instruments aimed at measuring the provision of person-centred practice among health care professionals across a range of settings. Edvardsson and Innes [4] found that many assessment tools relating to person-centred practice also failed to map on to theoretical frameworks. Harding, Wait and Scrutton [9] argue that there is a need to develop a strong evidence base that demonstrates the effectiveness of person-centred care and that such an evidence base needs to be informed by a theoretical person-centred framework.

Recently Slater, McCane and McCormack [10] published the development and psychometric properties of a holistic instrument for measuring person-centred health care based on the established person-centred practice theoretical framework of McCormack and McCance [1]; the Person-centred Practice Inventory – Staff (PCPI-S), that aims to measure the provision of person-centred practice across a range of settings. As this instrument does not exist in Norwegian, this article concerns the translation, cultural adaption and psychometric testing of this new instrument into Norwegian language.

### Description of the PCPI-S

The *PCPI-S* is a new instrument developed for health care staff in all health care settings that is theoretically aligned with McCormack & McCance’s person-centred practice framework. It consist of 59 statements (items) and uses a 5-point-Likert scale on all items except those concerning background data, with the following response categories: strongly disagree – disagree – neutral – agree – strongly agree. The 59 items pertain to constructs within the theoretical framework as depicted in Table 1. The items were derived from a consensus based process with experts on person-centredness described by Slater et al. [2]. The PCPI-S has been tested for validity and reliability and found to be psychometrically sound and aligned with the measurement model [2].

**Table 1:** Constructs within the PCPI-S with corresponding number of items

Constructs	No. of items in questionnaire
*Prerequisites*	
Professionally competent	3
Developed interpersonal skills	4
Being committed to the job	5
Knowing self	3
Clarity of beliefs and values	3
*Care environment*	
Skill-mix	3
Shared decision-making systems	4
Effective staff relationships	3
Power sharing	4
Potential for innovation and risk taking	3
The physical environment	3
Supportive organisational systems	5
Working with patients belief and values	4
*Care processes*	
Shared decision-making	3
Engagement	3
Having sympathetic presence	3
Providing holistic care	3
Total no. of items	59

## Aim

The aim of this paper is to translate, culturally adapt into Norwegian and test psychometrically, a measure of staff perceptions of the existence of a person-centred culture.

## Method

### Design

The study used a two-stage research design involving: translation and cultural adaption of the PCPI-S from English to Norwegian language (phase 1), and a quantitative cross sectional survey following psychometric evaluation (phase 2).

#### Phase 1: Translation and cultural adaption

The translation and cultural adaption of the PCPI-S follows the principles of good practice in translation and cultural adaptation, as laid out by Translation and Cultural Adaptation group of International Society For Pharmacoeconomics and Outcomes Research’s Quality of Life Special Interest group [11]. The steps recommended in a translation and cultural adaption are:

1. Preparation: initial work carried out before the translation work begins
2. Forward translation: translation from English to Norwegian by two independent translators
3. Reconciliation: comparing and merging two forward translations into a single forward translation
4. Back translation: translation of the new Norwegian language version back into the original English version by two independent translators
5. Back translation review: comparison of the back-translated versions of the instrument with the original to highlight and investigate discrepancies
6. Harmonization: comparison of back translations with multiple language versions
7. Cognitive debriefing: testing the instrument with a small group of relevant respondents
8. Review of cognitive debriefing results: comparison of the respondents’ interpretation of the translation with the original version to highlight and amend discrepancies
9. Proofreading: review to highlight and correct typographic, grammatical or other errors

According to the recommendations of the Translation and Cultural Adaptation group [11] the following roles took part in the process: project manager, instrument developer/independent translator, Key in-country consultant, forward translators, and back translators.

#### Phase 2: Psychometric evaluation

This phase examined the relationship of items to constructs by means of Confirmatory Factor Analysis in comparison with the psychometric evaluation performed on the original version of the PCPI-S, using the software packages SPSS 23.0 and Mplus 7. A quantitative cross sectional survey was used to generate data to test the measurement model as found in the original version of the PCPI-S [10]. The PCPI-S Norwegian version was tested with a convenience sample of nursing staff working in nursing homes, home care services or acute care settings in community health care in five Norwegian municipalities, and nursing staff working in one acute care ward at a large Norwegian hospital. With consent from the ward managers, a gate keeper in each setting distributed access to an online questionnaire or paper versions of the questionnaire, based on preferences discussed with each gate keeper/manager. All nursing staff in the different settings were invited and encouraged to participate by the gatekeepers, which included registered nurses, physicians, assistant nurses and assistants (without formal qualifications). The sample was chosen to achieve variation in practice settings, qualifications and length of experience. The data collection took place from May to October 2016. A total of 517 staff had the possibility to participate, which resulted in a response rate of 49.9% (*N* = 258). This produced a confidence interval of ±6.02% (based on population size = 10,000, confidence level 95% and anticipated percentage frequency = 50%). The ratio of respondent to item is important in factor analysis and Nunnally [12] recommends at least 10:1. Therefore the 59 items were partitioned and analysed according to concepts (Prerequisites; care environment and care Processes) in order to maximise the item to respondent ratio.

### Statistical analysis

In order to compare the Norwegian version with the original version of the PCPI-S, Confirmatory Factor Analysis was used to examine the theoretical measurement model. Missing data analysis for all 59 items showed all items to have negligible (less than 2%) and missing completely at random (Little’s MCAR test: chi square = 2036, df = 1999, sig = 2.73). Missing values were identified and included in the analysis. Many of the items had skewness scores indicating the presence of skewness and kurtosis (see Table 2) for items with skewness/kurtosis greater than ±2). Therefore, the data were analysed using Confirmatory Factor Analysis, whereby the original model was tested using Maximum Likelihood Robust (MLR) extraction as relevant with continuous and skewed data and a Orthogonal rotations (Varimax) to produce a clear factor structure. A factor loading of greater than 0.3 was acceptable [13] in keeping with guidelines on sample size. Minor modifications to the original model to improve model specification were permitted on the following ordering: (1) correlated errors across items within factors and (2) correlated errors across items across factors and (3) cross factor loadings of items to factors. The model was refined through a continuous and iterative process until it was considered acceptable, supported by a Root Mean Square Estimations of Approximation (RMSEA) of 0.06 or below; 90% RMSEA higher bracket below 0.08 [14, 15]; and Confirmation Fit Indices (CFI) of 0.90 or higher indicating good fit [14].

**Table 2:** Mean scores, measures of distribution and factor loadings of each item of the PCPI-Staff

Construct Scores and Items	Mean (Sd)	Skewness Kurtosis	Loading (Standard Error)
*Professionally Competent (Cronbach’s alpha)*			0.320
V1. I have the necessary skills to negotiate care options.	3.76 (0.75)	−.498 .520	0.308 (0.109)
V2. When I provide care I pay attention to more than the immediate physical task.	4.21 (0.81)	−1.682 4.84	0.329 (0.090)
V3. I actively seek opportunities to extend my professional competence.	4.15 (0.77)	−.832 1.01	0.457 (0.090)
*Developed Interpersonal Skills (Cronbach’s alpha)*			.727
V4. I ensure I hear and acknowledge others perspectives.	4.33 (0.54)	−.102 .200	0.659 (0.044)
V5. In my communication I demonstrate respect for others.	4.50 (0.56)	−.552 −.741	1.206 (0.275)
V6. I use different communication techniques to find mutually agreed solutions.	4.06 (0.67)	−.142 −.513	0.563 (0.053)
V7. I pay attention to how my non-verbal cues impact on my engagement with others.	4.10 (0.67)	−.342 0.85	0.588 (0.048)
*Being Committed to the Job (Cronbach’s alpha)*			0.668
V8. I strive to deliver high quality care to people.	4.55 (0.63)	−1.76 5.22	0.429 (0.120)
V9. I seek opportunities to get to know the person and their family in order to provide holistic care.	3.93 (0.77)	−.255 −.440	0.529 (0.071)
V10. I go out of my way to spend time with people receiving care.	3.56 (0.93)	−.410 .023	0.356 (0.090)
V11. I strive to deliver high quality care that is informed by evidence.	4.39 (0.67)	−1.205 2.789	0.578 (0.075)
V12. I continuously look for opportunities to improve the care experiences.	4.22 (0.69)	−1.195 5.087	0.625 (0.074)
*Knowing Self (Cronbach’s alpha)*			0.678
V13. I take time to explore why I react as I do in certain situations.	3.98 (0.69)	−.479 .523	0.681 (0.053)
V14. I use reflection to check out if my actions are consistent with my ways of being.	3.97 (0.70)	−.298 −.043	0.764 (0.054)
V15. I pay attention to how my life experiences influence my practice.	4.10 (0.66)	−.274 −.102	0.487 (0.062)
*Clarity of Beliefs and Values (Cronbach’s alpha)*			.638
V16. I actively seek feedback from others about my practice.	3.41 (0.90)	−.233 −1.69	0.504 (0.085)
V17. I challenge colleagues when their practice is inconsistent with our team’s shared values and beliefs.	3.37 (0.82)	−.064 −.176	0.678 (0.063)
V18. I support colleagues to develop their practice to reflect the team’s shared values and beliefs.	3.83 (0.67)	−.185 .004	0.705 (0.063)
*Skill-Mix(Cronbach’s alpha)*			0.456
V19. I recognise when there is a deficit in knowledge and skills in the team and its impact on care delivery.	4.04 (0.64)	−.307 .443	0.312 (0.076)
V20. I am able to make the case when skill mix falls below acceptable levels.	3.87 (0.81)	−.341 −.109	0.334 (0.167)
V21. I value the input from all team members and their contributions to care.	4.24 (0.70)	−.912 1.771	0.381 (0.150)
*Shared Decision-making Systems (Cronbach’s alpha)*			0.706
V22. I actively participate in team meetings to inform my decision-making.	3.47 (0.88)	−.031 −.236	0.618 (0.067)
V23. I participate in organisation-wide decision-making forums that impact on practice.	2.97 (1.05)	.097 −.561	0.649 (0.050)
V24. I am able to access opportunities to actively participate in influencing decisions in my directorate/division.	3.37 (1.09)	−.313 −.660	0.771 (0.047)
V25. My opinion is sought in clinical decision-making forums (e.g ward rounds, case conferences, discharge planning).	3.57 (0.88)	−.456 .097	0.453 (0.068)
*Effective Staff Relationships (Cronbach’s alpha)*			0.786
V26. I work in a team that values my contribution to person-centred care.	3.94 (0.72)	−.866 2.395	0.592 (0.068)
V27. I work in a team that encourages everyone’s contribution to person-centred care.	3.91 (0.75)	−.570 .681	0.723 (0.056)
V28. My colleagues positively role model the development of effective relationships.	4.02 (0.68)	−.406 .388	0.713 (0.051)
*Power Sharing (Cronbach’s alpha)*			0.718
V29. The contribution of colleagues is recognised and acknowledged.	3.79 (0.74)	−.468 .214	0.553 (0.061)
V30. I actively contribute to the development of shared goals.	3.73 (0.76)	−.392 .291	0.532 (0.061)
V31. The leader facilitates participation.	3.75 (0.81)	−.211 −.417	0.686 (0.042)
V32. I am encouraged and supported to lead developments in practice.	3.2 (0.93)	.002 −.298	0.708 (0.042)
*Potential for Innovation and Risk Taking (Cronbach’s alpha)*			0.523
V33. I am supported to do things differently to improve my practice.	3.25 (0.89)	−.019 −.062	0.735 (0.059)
V34. I am able to balance the use of evidence with taking risks.	3.44 (0.87)	−.560 .565	0.314 (0.081)
V35. I am committed to enhancing care by challenging practice.	3.78 (0.73)	−.324 .387	0.398 (0.072)
*The Physical Environment (Cronbach’s alpha)*			0.617
V36. I pay attention to the impact of the physical environment on people’s dignity.	4.20 (0.62)	−.357 .408	0.481 (0.071)
V37. I challenge others to consider how different elements of the physical environment impact on person-centredness (e.g. noise, light, heat etc).	3.36 (0.78)	−.078 −.017	0.672 (0.065)
V38. I seek out creative ways of improving the physical environment.	3.49 (0.75)	−.141 −.024	0.642 (0.072)
*Supportive Organisational Systems (Cronbach’s alpha)*			0.777
V39. In my team we take time to celebrate our achievements.	2.91 (0.93)	−.67 −.206	0.550 (0.062)
V40. My organisation recognises and rewards success.	3.06 (0.91)	−.085 −.029	0.644 (0.058)
V41. I am recognised for the contribution that I make to people having a good experience of care.	3.58 (0.86)	−.648 .780	0.719 (0.046)
V42. I am supported to express concerns about an aspect of care.	3.65 (0.79)	−5.67 .405	0.531 (0.069)
V43. I have the opportunity to discuss my practice and professional development on a regular basis.	3.60 (0.92)	−.459 −.264	0.619 (0.054)
*Working with Patients Belief and Values (Cronbach’s alpha)*			0.660
V44. I integrate my knowledge of the person into care delivery.	3.96 (0.62)	−.267 .477	0.585 (0.059)
V45. I work with the person within the context of their family and carers.	4.12 (0.61)	−.176 .046	0.560 (0.059)
V46. I seek feedback on how people make sense of their care experience.	3.42 (0.96)	−.157 −.443	0.520 (0.062)
V47. I encourage people to discuss what is important to them.	3.92 (0.73)	−.543 .785	0.678 (0.043)
*Shared Decision-making (Cronbach’s alpha)*			0.541
V48. I include the family in care decisions where appropriate and/or in line with the person’s wishes.	4.05 (0.68)	−.677 1.708	0.633 (0.066)
V49. I work with the person to set health goals for their future.	3.60 (0.84)	−.511 .642	0.688 (0.059)
V50. I enable people receiving care to seek information about their care from other healthcare professionals.	3.48 (0.89)	−.632 1.01	0.333 (0.077)
*Engagement (Cronbach’s alpha)*			0.755
V51. I try to understand the person’s perspective.	4.18 (0.59)	−1.13 9.241	0.738 (0.065)
V52. I seek to resolve issues when my goals for the person differ from theirs perspectives.	3.93 (0.68)	− 1.03 4.72	0.668 (0.074)
V53. I engage people in care processes where appropriate.	4.02 (0.71)	−1.31 5.57	0.689 (0.063)
*Having Sympathetic Presence (Cronbach’s alpha)*			0.776
V54. I actively listen to people receiving care to identify unmet needs.	4.16 (0.65)	−1.11 5.80	0.847 (0.038)
V55. I gather additional information to help me support people receiving care.	3.84 (0.74)	−.956 3.05	0.680 (0.054)
V56. I ensure my full attention is focused on the person when I am with them.	4.22 (0.61)	−1.19 7.75	0.716 (0.082)
*Providing holistic care (Cronbach’s alpha)*			0833
V57. I strive to gain a sense of the whole person.	3.86 (0.87)	−.867 1.64	0.692 (0.062)
V58. I assess the needs of the person, taking account of all aspects of their lives.	3.65 (0.85)	−.435 .586	0.704 (0.046)
V59. I deliver care that takes account of the whole person.	4.21 (0.71)	−1.186 4.45	0.863 (0.048)

## Results

The translation and cultural adaption was carried out according to the recommendations as laid out by Wild, Grove, Martin, Eremenco, McElroy, Verjee-Lorenz and Erikson [11]. The following is a detailed description of the work carried out throughout the nine recommended steps.

*1. Preparation*: initial contact with the developer of the PCPI-S was made, and permission for translation from English into Norwegian language was obtained. The instrument developer agreed to be involved in the process.

*2. Forward translation*: two independent forward translations from English to Norwegian language were performed. Both forward translators were fluent speakers of the target language (Norwegian) and the original language (English).

*3. Reconciliation*: the two forward translators compared and merged the forward translations into one single forward translation. The reconciliation sought to resolve discrepancies between the forward translations as e.g. individual speech habits and preferences, and thus alternative translations were produced in the final reconciled forward translation ready for back translation.

*4. Back translation*: two independent back translations from Norwegian to English language were performed. Both back translators were fluent speakers of the original language (English) and of the target language (Norwegian). The back translation had a focus on a conceptual translation as opposed to a more literal back translation, as the concepts of the PCPI-S are important to maintain in the process.

*5. Back translation review*: the purpose of the backward translation process is to ensure that the same meaning can be derived when the translation is moved back into the original language. First, the two back translators compared and merged the back translations into one single back translation. Then, the single back translation was reviewed by the instrument developer against the original version. He addressed problematic translations in order to ensure conceptual equivalence of the translation. The discrepancies were addressed and revised by all translators until consensus was reached on a reconciled version of the translation. Examples of concepts that were worked on with comments from the instrument developer are:

- Patient: “we avoid the use of ‘patients’ as it is not a person-centred term and not all service users are patients”.
- Ward: “I have a problem with the continuous use of the word ‘ward’ as opposed to team, as it will really limit the use of the instrument in other settings in Norwegian healthcare - settings that aren’t hospital wards, such as clinics, community settings etc.”
- Nursing: “The PCPI-S is not just nursing focused so this needs to be ‘care’ or ‘practice’”.

*6. Harmonisation*: is a step in the process that is applicable when there are several cross-cultural translations of an instrument available. Harmonisation across different translations is performed to ensure cross-cultural validity, and thus comparable psychometric evaluations. The instrument developer is not aware of any other translations and harmonisation was therefore not applicable in this process, but might be at a later stage.

*7. Cognitive debriefing*: concerns testing the instrument on a small group of relevant respondents. The Norwegian version of the PCPI-S was tested by five nurses with different length of experience, and with little or no familiarity of the philosophy person-centred health care.

*8. Review of cognitive debriefing results and finalization*: The key in-country consultant sat down with each of the five nurses in order to test alternative wording and to check understandability, interpretation, and cultural relevance of the translation. Only a few minor grammatical revisions were made after the cognitive debriefing.

*9. Proofreading*: was performed by the key in-country consultant, the project manager and one of the translators to correct typographic, grammatical or other errors.

### Demographic details of sample

The sample consisted of 88% women and 12% men, of which the mean length of work experience was 10.4 years (Range 1–45). Most respondents were RNs (62%), followed by assistant nurses (19.8%), assistants (6.6%) and physicians (2.7%).

### Instrument testing

All 59 items were positively scored with mean scores ranging from 3.03 (item 40) to 4.83 (item 8). Skewness was not a significant issue across the 59 items and kurtosis was noted in 9 items (See Table 2) however these scores were low and contrary to measures of skewness. Examination of the correlation matrix shows scores ranged from nonsignificant relationships of − 0.004 to 0.627 (*p* = 0.01). No relationship between items breached collinearity. Examination of the Kaiser Meyer Olkin measure of sampling adequacy = 0.838; Bartletts test for Spericity (app Chi-square = 6220, df = 1711, sig = 0.00) indicated the appropriateness of the data for Confirmatory Factor Analysis.

A sample of 258 staff (49.9%) out of a potential sample of 517 staff drawn from five municipalities and one hospital completed the 59 items of the PCPI-S. In order to present a sufficient respondent to item ratio [13] for the factor analysis the items were analysed according to item structure across concepts (Prerequisites 18 items: 14:1 ratio; Care Environment 26 items 9:1 ratio; Care Processes 15 items: 17: 1 ratio). Hair, Black, Babib, Anderson and Tatham [13] recommend with a sample above of 250 participants the factor loading of 0.35 be used (p.112). Cronbach’s alpha scores were judged by < 0.4 = unacceptable; 0.5 ≤ α < 0.6 Poor; 0.6 ≤ α < 0.7 Acceptable; 0.7 ≤ α < 0.9 Good; α ≥ 0.9 Excellent [16].

### Modifications to the model

#### Prerequisites

Modifications to the model; Correlated error between items V10 with V9 (28.792); V11 and V8 (35.923); V10 with V1 (13.105); V5 with V11 (11.112). Cross factor loading V5 on Knowing self (15.219).

#### The care environment

Modifications to the model; Correlated error between items V40 and V39 (46.367); V33 with V32 (25.289); V29 with V28 (15.781); V26 with V25 (14.649); V21 with V19 (12.515); V43 with V42 (11.171); V30 with V22 (11.072).

#### The care process

Modifications to the model; Correlated error between items V58 and V57 (17.412); V51 with V50 (13.319) Table 3.

**Table 3:** Fit Statistics for alternative measurement models of the PCPI-Staff

Models	RMSEA	90% RMSEA	CFI	TLI	SRMR
Prerequisites	0.048	0.035–0.061	0.914	0.89	0.055
The Care Environment	0.052	0.043–0.061	0.898	0.876	0.063
Care Processes	0.056	0.042–0.069	0.938	0.919	0.052

### Summary of psychometric findings

Cronbach alpha scores for each of the constructs are included in Table 2. Thirteen of the 17 constructs had alpha scores greater than 0.6 and deemed an acceptable score [16]. Four of the factors had Cronbach alpha scores that were lower than 0.6. Examination of the factor items show that each of these factors contained items that failed to achieve acceptable factor loadings (items 1, 2, 19, 20, 34, 50). However, these were retained in the overall instrument as they were statistically significant and contributed to the model fit. One item (v5) was included as a cross factor loading and examination of the loading scores show that the item relationship is significant on both factors, however it was maintained on its original factor as it had a greater contribution to the factor. Cronbach alpha scores for Person-centred Practice Inventory = .933; Prerequisites = .806; Care environment = .891, Care process = .888). All scores are high and excellent.

## Discussion

### Translation and cultural adaption

Poor translation methods can present risk to research data. The quality of data derived from translated measures, and thus the conclusions drawn from them, relies on the accuracy and quality of the translation. As a response to the many methods for instrument translation with varying quality available, the Translation and Cultural Adaptation group of International Society For Pharmacoeconomics and Outcomes Research’s Quality of Life Special Interest group published their consensus on principles of good practice in translation and cultural adaptation [11]. The translation and cultural adaption of the PCPI-S follows these recommended steps in translation and cultural adaptation with the aim of establishing a Norwegian instrument that is conceptually equivalent to the original.

The translation and cultural adaption process of questionnaires can be a complex and demanding process. The elements of language should not be underestimated. Key concepts like care, competence and services can be challenging to translate given the meaning connected to different languages, cultures, and professional contexts. The PCPI-S should preferably be applicable to several professional contexts, and not be limited to nursing. We found it challenging to translate the PCPI-S to assure interdisciplinary applicability and appropriateness. The most challenging concept to translate from English was “care”, as the direct Norwegian translation of this concept is affiliated to nursing mostly, and is not a concept that e.g. physicians or physiotherapist affiliate with. As the PCPI-S is a questionnaire that aims at investigating person-centred health care across professions, the translation of the concept must be deliberate and appropriate for all disciplines meant to be investigated. Wild, Grove, Martin, Eremenco, McElroy, Verjee-Lorenz and Erikson [11] support this by stating that important components in the process are clear explanation of the basic concepts, with the intention that the translation will capture the conceptual meaning of the items rather than being a literal translation. This has to be balanced with producing colloquial translations that will be easily understood by the general lay population or intended respondents.

The inclusion of the instrument developer in the translation and cultural adaption process was vital in this regard. The instrument developer is both the developer of the underpinning theory as well as the measurement instrument, and is therefore the key expert on the concepts and meanings meant to be conveyed in the PCPI-S. The instrument developer was able to clarify the concepts behind the translated items and how the first back translations did not convey the original meaning, as well as ambiguities and misinterpretation of items. As expressed by the Translation and Cultural Adaptation group, the inclusion of the instrument developer in the back translation review is one of the most important components of the cross-cultural adaptation process [11], but one that most of the existing guidelines had not specifically addressed. Reconciliation and harmonisation of a translated version with the original version of an instrument has the objective of achieving the same conceptual meaning between the two in order to maintain the same psychometric performance and thus derive data that is comparable. The development of standardised cross-cultural tools facilitates the accumulation of internationally comparable data as well as providing a strong evidence-base, in this case for staff perceptions of person-centred Practice.

### Psychometric properties

Overall, the psychometric properties of the tool were acceptable. The model fit statistics were overall positive (RMSEA .0.06, of 0.06 or below; 90% RMSEA higher bracket below 0.08 and Confirmation Fit Indices (CFI) of 0.90 or higher); the model requires minor modifications and these are mostly confined to correlated errors. There was only one cross factor loading included in the model. The skewness and kurtosis scores of a number of items were of concern but overall these did not impact on the factor model and it was maintained. There were some areas that could be improved but further testing with larger samples would help clarify these issues. All items were scored positively indicating that participants felt that they provided person-centred care.

### Choice of instrument

There are numerous instruments available that measure person-centred health care as found by The Health Foundation [5] but it remains the case that the majority of these are proxy measures. Apart from this translation, there are at least two other instruments concerning staff’s view of person-centredness already translated into Norwegian: the Person- centred Climate Questionnaire–Staff version [7], and the Person-centred Care Assessment Tool [8]. So when choosing an instrument for research purposes the conceptual underpinnings of the tool needs consideration in relation to the wider application of the tool. Edvardsson and Innes [4] found that many person-centred practice measurement tools are not supported by or located within theoretical frameworks. The PCPI-S addresses the wider principles of person-centred practice outlined above by McCormack and McCance [1], and is based on the established person-centred practice theoretical framework.

The tools that are available vary in the perspectives studied; some look at staff, some look at family caregivers, while most take the perspective of the person receiving health care [4]. This reflects research interest in engaging with the range of key stakeholders involved in health care, both care recipients and caregivers, which is in line with the philosophy of person-centredness. The multitude of instruments and conceptual underpinnings available might be a reflection of different conceptual language in different care settings, but one might ask if the differences between settings are of such nature that they cannot be investigated with one instrument. The person-centred practice framework encompass all setting where health care is provided. The fact that the PCPI-S measures the provision of person-centred practice among health care professionals across a range of settings is an important strength of the instrument, and few existing instruments have this generic applicability [5].

### Limitation

The sample in this study is a convenience sample, meaning that further validity testing in a Norwegian setting should be performed on a representative sample in order to generalize the results further. This study made use of Confirmatory Factor Analysis as a means of psychometric analysis, but other analyses like a test-retest procedure and concurrent validity against another instrument measuring person-centred practice should be the next steps in further psychometric validation of the PCPI-S, both in the Norwegian but also other translations and cultural adaptions. Moreover, there are items that require further testing, and the full instrument should be tested as a 17 constructs measurement model. This would require a larger sample size. Correlated errors were statistically significant and permitted. Examination of the theoretical framework show it contains macro element that impact on all factors contained in the PCPI-Staff. This fact and the fact that the sample size limited the examination of the full instrument, may have contributed to the correlated errors.

## Conclusion

The translation and cultural adaption process of the PCPI-S from English to Norwegian language was a demanding process in order to retain the conceptual meanings of the original instrument. Overall, the psychometric properties of the tool were acceptable, but testing on a larger, representative sample is recommended. Strengths of the PCPI-S are that it maps onto a recognised theoretical person-centred framework, as well as it is developed for and tested in various health care settings. The Norwegian version of the PCPI-S allows exploration of the attributes of staff working within complex organisational systems, in order to provide effective care through the person-centred processes. Choosing one instrument to study a variety of setting is necessary to produce a strong evidence base of the effectiveness of person-centred health care.

## Additional file

Additional file 1: Dataset for testing of PCIP Staff. SPSS original dataset for psychometric analyses. (SAV 32 kb)

## Abbreviations

CFI: Confirmation Fit Indices
MLR: Maximum Likelihood Robust
PCPI-S: Person-centred Practice Inventory – Staff
RMSEA: Root Mean Square Estimations of Approximation
